# Readability and Information Quality in Cancer Information From a Free vs Paid Chatbot

**DOI:** 10.1001/jamanetworkopen.2024.22275

**Published:** 2024-07-26

**Authors:** David Musheyev, Alexander Pan, Preston Gross, Daniel Kamyab, Peter Kaplinsky, Mark Spivak, Marie A. Bragg, Stacy Loeb, Abdo E. Kabarriti

**Affiliations:** 1 Department of Urology, State University of New York Downstate Health Sciences University, New York; 2 Department of Urology, New York University and Manhattan Veterans Affairs, New York; 3 Marketing Department, Stern School of Business, New York University, New York; 4 Department of Population Health, New York University, New York

## Abstract

**Importance:**

The mainstream use of chatbots requires a thorough investigation of their readability and quality of information.

**Objective:**

To identify readability and quality differences in information between a free and paywalled chatbot cancer-related responses, and to explore if more precise prompting can mitigate any observed differences.

**Design, Setting, and Participants:**

This cross-sectional study compared readability and information quality of a chatbot’s free vs paywalled responses with Google Trends’ top 5 search queries associated with breast, lung, prostate, colorectal, and skin cancers from January 1, 2021, to January 1, 2023. Data were extracted from the search tracker, and responses were produced by free and paywalled ChatGPT. Data were analyzed from December 20, 2023, to January 15, 2024.

**Exposures:**

Free vs paywalled chatbot outputs with and without prompt: “Explain the following at a sixth grade reading level: [nonprompted input].”

**Main Outcomes and Measures:**

The primary outcome measured the readability of a chatbot’s responses using Flesch Reading Ease scores (0 [graduate reading level] to 100 [easy fifth grade reading level]). Secondary outcomes included assessing consumer health information quality with the validated DISCERN instrument (overall score from 1 [low quality] to 5 [high quality]) for each response. Scores were compared between the 2 chatbot models with and without prompting.

**Results:**

This study evaluated 100 chatbot responses. Nonprompted free chatbot responses had lower readability (median [IQR] Flesh Reading ease scores, 52.60 [44.54-61.46]) than nonprompted paywalled chatbot responses (62.48 [54.83-68.40]) (*P* < .05). However, prompting the free chatbot to reword responses at a sixth grade reading level was associated with increased reading ease scores than the paywalled chatbot nonprompted responses (median [IQR], 71.55 [68.20-78.99]) (*P* < .001). Prompting was associated with increases in reading ease in both free (median [IQR], 71.55 [68.20-78.99]; *P* < .001)and paywalled versions (median [IQR], 75.64 [70.53-81.12]; *P* < .001). There was no significant difference in overall DISCERN scores between the chatbot models, with and without prompting.

**Conclusions and Relevance:**

In this cross-sectional study, paying for the chatbot was found to provide easier-to-read responses, but prompting the free version of the chatbot was associated with increased response readability without changing information quality. Educating the public on how to prompt chatbots may help promote equitable access to health information.

## Key Points

**Question:**

Does a chatbot perpetuate inequity for patients’ access to consumer health information about cancer through poor readability and paywalls?

**Findings:**

In this cross-sectional study of 100 chatbot-generated responses, a paid chatbot was associated with easier-to-read responses than the free version of the chatbot but prompting the free version to reword responses at a sixth grade reading level was associated with increased readability to match the paid version. Information quality was similarly high across different models.

**Meaning:**

These findings suggest that a chatbot can exacerbate health information inequities, but precise prompting is associated with increased readability without compromising information quality.

## Teaser

This cross-sectional study examines the readability and quality differences in cancer information found on free and paywalled versions of a chatbot.

## Introduction

Health disparities are pervasive in the screening, diagnosis, and treatment of cancer, which are associated with negative clinical outcomes.^1,2,3^ For example, patients with low socioeconomic status have poorer cancer care and outcomes than patients with higher income.^1^ Health literacy may be key in addressing these disparities.^4,5^ Patients with higher health literacy are more likely to adhere to screening protocols for breast, cervical, and colorectal cancer than patients with lower health literacy.^3^ One study showed that patients with prostate cancer had an increased likelihood to regret their treatment(s) if they demonstrated lower health literacy.^6^ Low health literacy is especially concerning in environments where patients encounter inaccessible or inaccurate information, physician shortages, and language or cultural differences.^3^ For this reason, the American Medical Association recommends patient-facing health information to be written at a sixth grade or lower reading level.^7^

The emergence of consumer-facing large language models (LLMs) enables patients to now use chatbots to access health-related information. While the use of chatbots for seeking health-related information has become increasingly promising in recent years,^8^ considering the downstream impact of these chatbots and their implications for health equity is important. Consumer health care information is often not individualized to patients’ literacy levels.^9^ A recent study^10^ found artificial intelligence (AI) outputs propagating biased health care information associated with gender, race, ethnicity, and socioeconomic status. Other studies^11,12,13^ have shown AI chatbot responses to be presented above recommended reading levels for consumer health information, which could limit patients’ abilities to understand information and take appropriate action for their care.

One paywalled chatbot requires a $20 per month subscription, presenting another accessibility barrier for low-income individuals who may benefit from the additional knowledge and reasoning capabilities provided by the paid version. Given the cost and performance differences between the free and paywalled versions of the chatbot, this study aimed to evaluate the quality and readability of the 2 versions’ responses to common queries about cancer. We sought to determine if inequities exist in AI by investigating whether the paywalled chatbot provided easier-to-read responses compared with the free version and whether prompts could address any observed differences in readability and quality of cancer information.

## Methods

This cross-sectional study followed the Strengthening the Reporting of Observational Studies in Epidemiology (STROBE) reporting guideline.^14^ The study was determined exempt from review and the requirement of informed consent by the SUNY Downstate Health Sciences University institutional review board.

The 5 types of cancer with the highest projected mortality include lung, breast, prostate, colorectal, and skin cancers and were used as search terms in this study.^15^ The top 5 Google Trends queries associated with search terms *lung cancer*, *breast cancer*, *prostate cancer*, *colorectal cancer*, and *skin cancer *were used as chatbot inputs and were collected on December 1, 2023. The search tracker was used to identify publicly searched cancer terms and its settings were: US location, time range of January 1, 2021, to January 1, 2023, all search categories, and web-based searches (eTable 1 in Supplement 1).

Using 25 search-related queries, 100 individual outputs were generated with and without prompting in the free chatbot (ChatGPT version 3.5, Open AI) and paywalled chatbot (ChatGPT version 4.0, OpenAI). To ensure independence of responses, new chatbot conversations were initiated following each response generation. First, search queries from the search tracker were inputted into versions 3.5 and 4.0 of the chatbot and responses were obtained (eTable 2 in Supplement 1). Afterwards, responses were prompted with the following: “Explain the following at a sixth grade reading level: [nonprompted input]”.

Trained researchers (A.P. and D.M.), blinded to the chatbot model and input prompting, evaluated responses using the validated DISCERN questionnaire. DISCERN functions to rate the quality of health information for average health care consumers from a variety of information sources.^16^ The graders reported DISCERN scores as the mean of the 15 questions in the questionnaire rounded to the nearest whole number to represent the overall DISCERN score which was considered to be DISCERN’s sixteenth question score. The rounded whole numbers were used to find the mean among their groups (chatbot version 3.5 prompted, chatbot version 3.5 nonprompted, chatbot version 4.0 prompted, and chatbot version 4.0 nonprompted) to get a total of 100 unique scores. Interrater variability was calculated between evaluator DISCERN scores using a 2-way random-effects intraclass correlation for agreement.

### Statistical Analysis

To evaluate response readability, Flesch-Kincaid Reading Ease and Grade Level were calculated for each output (eTable 3 in Supplement 1).^17,18^ Values were compared pairwise between the free and paywalled chatbot, both prompted and unprompted response, using a 2-sided paired *t* test with Benjamini-Hochberg adjustment for multiple comparisons for a total of 6 unique *t* tests. *P* < .05 was considered significant. Data analyses were done in R version 4.3.2 (R Project for Statistical Computing). Analyses were performed from December 20, 2023, to January 15, 2024.

## Results

Nonprompted free chatbot responses had significantly worse readability (median [IQR] Flesch Reading ease score, 52.60 [44.54-61.46]) compared with nonprompted paywalled chatbot (median [IQR], 62.48 [54.83-68.40]; *P* = .02). Compared with the nonprompted responses, the prompted free chatbot (median [IQR], 71.55 [68.20-78.99]; *P* < .001), and the prompted paywalled chatbot (median [IQR], 75.64, [70.53-81.12]; *P* < .001) responses had significantly better readability. Finally, the nonprompted paywalled chatbot responses had significantly worse readability than the prompted paywalled chatbot responses (*P* < .001) (Figure). The Flesch Kincaid Grade level mean (SE) for the nonprompted free and paywalled chatbot were 11.6 (0.15) and 10.4 (0.09), respectively, while the prompted free and paywalled chatbot grade levels were 9.5 (0.07) and 8.4 (0.05), respectively (Figure).

**Figure.**
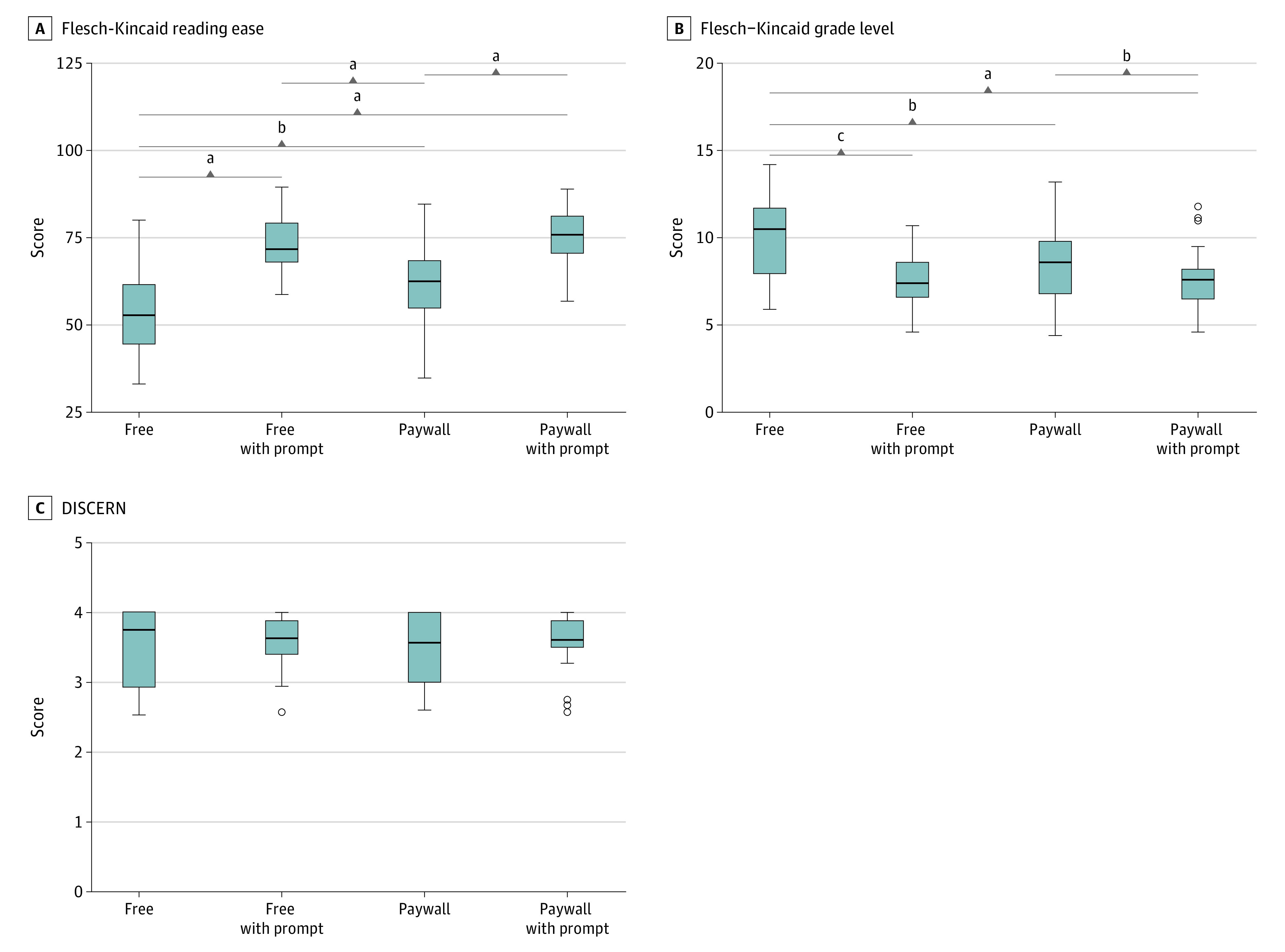
Readability and Quality Metrics of GPT−Generated Text Distributions for the readability and quality scores of text generated by the free and paywalled chatbots with and without prompting: (A) Flesch-Kincaid reading ease scores, scored from 0 (easy to read) to 100 (difficult to read); (B) Flesch-Kincaid grade level; (C) DISCERN scores, scored from 0 (low quality) to 5 (high quality). The lines within boxes indicate medians, the top and bottom of the boxes indicate IQRs, the whiskers indicate 95% CIs, and the circles indicate outliers. ^a^*P* < .001. ^b^*P* < .05. ^c^*P* < .01.

DISCERN interrater agreement was 93% (95% CI, 89.7-95.2; *P* < .001). The median (IQR) overall DISCERN grades of the nonprompted free chatbot version 3.5 was 3.8 (2.9-4.0), 3.6 (3.0-4.0) for the nonprompted chatbot version 4.0, 3.6 (3.4-3.0) for the prompted chatbot version 3.5, and 3.6 (3.5-3.9) for the prompted chatbot version 4.0, with no statistically significant differences among any of the output scores regardless of the chatbot version or use of prompts (Figure).

## Discussion

To our knowledge, this is the first study comparing the readability and quality of cancer information produced by free vs paid chatbot—with and without prompts. Increased readability was associated with a prompt for the free version. This could have implications for reducing patients’ access to reader-friendly information that could better empower them to gain knowledge and action steps for their own care and reduce potential disparities, such as adherence to cancer screening protocols or regret following treatment.^3,6^

This study also found comparable quality of consumer health information between free and paid versions of the chatbot with and without prompting. The association of increased readability with the chatbot’s ability to maintain consistent overall DISCERN scores (eg, median scores ranged from 3.6 to 3.8) is promising for the text simplification capabilities of LLMs. Simplifying text did not reduce the quality of health information in this study. The lowest subdomain DISCERN scores were in the categories involving references and treatment. To be more specific, the free and paid version of the chatbot did not cite their sources and treatment information lacked discussion of risks, benefits, and overall impact on quality of life. Fortunately, the chatbot scored high in subdomains specifying the aims of outputs and avoiding biases in its responses.

It is problematic that both the free and paid versions of the chatbot gave suboptimal readability scores (version 3.5: reading ease, 52.60; grade level, 11.6; version 4.0: reading ease, 62.48; grade level, 10.4) that exceed the recommended sixth grade reading level for consumer health information. Indeed, we found a median 12th grade reading material in the free chatbot responses about cancer, which is consistent with data from a previous study suggesting little change in the chatbot’s readability over time.^10^ A previous study found that the chatbot’s production of information in the subject of orthopedic pathologies was significantly more difficult to read than similar information on health-related websites.^19^ The results of this study suggest that prompting may help to minimize the issue of reading difficulty.

Our study suggests that prompting the chatbot to respond at a 6th grade reading level was associated with increased reading ease in both the free and paid chatbot versions, yielding 8th grade to 10th grade level responses (Figure). Therefore, an extra step prompting the chatbot to respond at a lower grade reading level can make health information more readable and further accessible for laypeople.

After prompting, the chatbot may have inappropriately interpreted its audience to be a sixth grader rather than providing a Flesch-Kincaid sixth-grade reading level response. For example, some prompted responses recommended discussing cancer issues with an adult or teacher in addition to a doctor, implying a flawed prompt interpretation. Despite prompting the chatbot to give responses at a sixth grade reading level, median responses were still at a higher grade level. These data are consistent with a previous study that prompted the chatbot to give information at a more readable level, but produced responses that were at the 10th grade level.^20^ Further investigation is warranted for identifying better chatbot prompts to improve information quality, increase readability and how to prompt chatbots to better understand human requests.

### Limitations

This study has limitations. We limited the study to using top search queries according to the search tracker’s data as opposed to the top searched queries submitted by the public to the paid and unpaid chatbot software because those data were not available to the public. Also, chatbot responses were not restricted to a set length. Stochasticity in response lengths can be a potential confounder for readability and information quality. Finally, our study did not address accuracy in prompted and nonprompted chatbot responses. A previous study^21^ found that chatbot produced accurate responses to 11 of 13 directed questions on common myths about cancer.

## Conclusions

These findings suggest that chatbots have the potential to contribute to cancer health inequities when responses are presented above the recommended sixth grade reading level for consumer health information, particularly when the general public is seeking cancer information with the unpaid version of a chatbot. However, prompting the chatbot to respond to common queries about cancer at a sixth grade reading level provides an associated increase in readability with no significant change in health information quality. This presents an opportunity for physicians to coach their patients on how to search for information on the chatbot at a more readable level.

## Notes

Supplement 1. **eTable 1.** Top 5 Queries of the Top 5 Most Common Cancers From January 1, 2021, to January 1, 2023, According to Google Trends **eTable 2.** Examples of the Free and Paid Chatbot Versions’ Responses to Prompted and Unprompted Query Inputs **eTable 3.** Interpretation of Flesch-Kincaid Reading Grade Levels and Formula for Reading Ease

Supplement 2.
Data Sharing Statement
