# Supplementary material for: Readability and Information Quality in Cancer Information From a Free vs Paid Chatbot
Source: JAMA Netw Open. 2024 Jul 26;7(7):e2422275. doi: 10.1001/jamanetworkopen.2024.22275 (PMC11282443; doi:10.1001/jamanetworkopen.2024.22275)
Supplement: Supplement 1. — eTable 1. Top 5 Queries of the Top 5 Most Common Cancers From January 1, 2021, to January 1, 2023, According to Google Trends eTable 2. Examples of the Free and Paid Chatbot Versions’ Responses to Prompted and Unprompted Query Inputs eTable 3. Interpretation of Flesch-Kincaid Reading Grade Levels and Formula for Reading Ease [file jamanetwopen-e2422275-s001.pdf]

Supplemental Online Content

Musheyev D, Pan A, Gross P, et al. Readability and information quality in cancer information from free vs paid ChatGPT. *JAMA Netw Open*. 2024;7(7):e2422275. doi:10.1001/jamanetworkopen.2024.22275

eTable 1. Top 5 Queries of the Top 5 Most Common Cancers From January 1, 2021, to January 1, 2023, According to Google Trends

eTable 2. Examples of Free and Paid Chatbot Versions' Responses to Prompted and Unprompted Query Inputs

eTable 3. Interpretation of Flesch-Kincaid Reading Grade Levels and Formula for Reading Ease

This supplemental material has been provided by the authors to give readers additional information about their work.

© 2024 Musheyev D et al. *JAMA Network Open.*

eTable 1. Top 5 Queries of the Top 5 Most Common Cancers From January 1, 2021, to January 1, 2023, According to Google Trends

	Skin cancer	Colorectal cancer	Prostate cancer	Lung cancer	Breast cancer
1	what is skin cancer	colorectal screening	prostate cancer symptoms	lung cancer symptoms	breast cancer symptoms
2	cancer symptoms	colorectal cancer screening	cancer symptoms	cancer symptoms	breast cancer awareness
3	skin cancer symptoms	symptoms colorectal cancer	prostate cancer treatment	what is cancer	what is breast cancer
4	skin cancer pictures	colon cancer	what is prostate	what is lung cancer	cancer symptoms
5	melanoma	colon	what is prostate cancer	small cell lung cancer	breast cancer signs

© 2024 Musheyev D et al. *JAMA Network Open.*

eTable 2. Examples of Free and Paid Chatbot Versions' Responses to Prompted and Unprompted Query Inputs

Input	Free non-prompted Chat GPT	Free prompted ChatGPT	Paid non-prompted Chat GPT	Paid prompted ChatGPT
colorectal cancer symptoms	The symptoms of colorectal cancer can vary depending on the location and stage of the cancer. Some common symptoms include:<br><br>1. Changes in bowel habits: Persistent diarrhea or constipation, or a change in the size, shape, or consistency of the stool.<br><br>2. Rectal bleeding: Blood in the stool, often bright red or very dark in color.<br><br>3. Abdominal discomfort: Cramps, pain, or a feeling of fullness or bloating in the abdominal area.<br><br>4. Unexplained weight loss: Losing weight without trying, and without any explanation.<br><br>5. Fatigue or weakness: Feeling tired or weak, even	Colorectal cancer is a type of cancer that affects the colon or rectum, which are part of our digestive system. When someone has colorectal cancer, they may experience certain symptoms that could indicate there might be something wrong.<br><br>One common symptom of colorectal cancer is changes in bowel habits. This means that a person might notice a difference in how often they go to the bathroom, or they might have diarrhea or constipation. They might also feel like they need to go to the bathroom but nothing comes out.<br><br>Another symptom is blood in the stool. This means that when a person uses the bathroom, they might see blood in the toilet or on the toilet paper. It's important to note that blood in the stool can also be caused by other factors, so it's always a good idea to speak with a doctor about this symptom.<br><br>Some people with colorectal cancer might experience abdominal pain, which is another symptom. This pain might feel	1. Changes in bowel habits, such as diarrhea, constipation, or narrowing of the stool, that lasts for more than a few days<br>2. A feeling that you need to have a bowel movement that is not relieved by doing so<br>3. Rectal bleeding or blood in your stool<br>4. Persistent abdominal discomforts, such as cramps, gas, or pain<br>5. A feeling of not being able to empty your bowel completely<br>6. Weakness or fatigue<br>7. Unexplained weight loss<br>8. Iron deficiency anemia (a low red blood cell count)<br><br>Note: Many people with colorectal cancer experience no symptoms in the early stages of the disease. Moreover, these symptoms can also be caused by conditions other than colorectal cancer, such as infection or hemorrhoids. Any persistent symptoms should be	Colorectal cancer symptoms are signs that someone might have a disease in their colon or rectum, which are parts of the body that help digest food. Just like when you have a cold, you might have symptoms like a runny nose or a cough, colorectal cancer also has its own symptoms. But, these symptoms aren't as easy to notice right away. They can include:<br><br>1. Wanting to poop more often than normal or having a hard time going poop.<br>2. Seeing blood in your poop.<br>3. Feeling very tired all the time.<br>4. Losing weight without trying.<br>5. Feeling like you're not completely empty after you poop.<br><br>Not everyone with colorectal cancer will have these symptoms. And just because someone has these symptoms, it doesn't always mean they have this type of cancer. These could be signs of other problems too. But if you or

© 2024 Musheyev D et al. *JAMA Network Open.*

Input	Free non-prompted Chat GPT	Free prompted ChatGPT	Paid non-prompted Chat GPT	Paid prompted ChatGPT
	with minimal physical activity.<br><br>6. Iron deficiency anemia: Low red blood cell count due to chronic bleeding, leading to symptoms such as fatigue, pale skin, and shortness of breath.<br><br>7. Narrow stools: Stools that are thinner than usual, which may indicate a blockage in the colon.<br><br>8. Bowel obstruction: Severe abdominal pain, vomiting, and the inability to pass stool or gas may indicate a bowel obstruction caused by advanced colorectal cancer.<br><br>It is important to note that these symptoms can also be caused by other conditions. If you experience any of these symptoms, it is recommended to consult a healthcare professional for a proper evaluation and diagnosis.	like cramps or discomfort in the stomach area. It can also be accompanied by bloating, which makes the stomach feel swollen.<br><br>Unintended weight loss can also be a symptom of colorectal cancer. This means losing weight without trying or making any changes to your diet or exercise routine. If someone is losing weight without a clear reason, it's worth mentioning to a doctor.<br><br>Lastly, feeling tired or weak all the time is another symptom of colorectal cancer. If someone is constantly feeling exhausted, even after getting enough sleep and rest, it could be a sign of something wrong with their body.<br><br>Remember, these symptoms do not automatically mean that someone has colorectal cancer. Many other conditions can cause similar symptoms. If any of these signs persist or if you're worried about your health, it's always best to talk to a doctor who can help figure out what might be going on.	evaluated by a healthcare professional.	someone else has these symptoms, it's important to tell a doctor so they can check what's going on.

© 2024 Musheyev D et al. *JAMA Network Open.*

Input	Free non-prompted Chat GPT	Free prompted ChatGPT	Paid non-prompted Chat GPT	Paid prompted ChatGPT
colon cancer	Colon cancer, also known as colorectal cancer, is a type of cancer that affects the colon or rectum. It occurs when abnormal growths, known as polyps, form in the lining of the colon or rectum and eventually become cancerous.<br><br>Risk factors for colon cancer include age, family history of the disease, personal history of certain types of polyps or inflammatory bowel disease, as well as lifestyle factors such as a diet high in red or processed meats, lack of physical activity, obesity, smoking, and heavy alcohol use.<br><br>Symptoms of colon cancer can vary and may include changes in bowel habits (such as diarrhea or constipation), blood in the stool, abdominal pain or cramping, weakness or fatigue, unintended weight loss, and a feeling that the bowel does not empty completely.	Colon cancer is a disease that happens in a part of your body called the colon, which is a long tube in your belly. It's a type of cancer, which means it's a serious illness that can make you really sick.<br><br>In our bodies, new cells are constantly being made and old cells die off. Sometimes, something goes wrong in this process, and cells start growing too much and not dying like they should. This is what happens in colon cancer.<br><br>Colon cancer usually starts as a small growth called a polyp on the wall of the colon. Over time, the cells in the polyp can turn into cancer cells. If the cancer cells are not caught and treated early, they can keep growing and spreading to other parts of the body.<br><br>The symptoms of colon cancer can vary, but some common signs include stomach pain, changes in bowel movements (like diarrhea or constipation), blood in the stool, and feeling tired all the time. It's important to tell a doctor if you are experiencing any of these symptoms.	Colon cancer is a type of cancer that originates in the cells of the colon or rectum, which are parts of the large intestine. Most cases of colon cancer begin as noncancerous polyps, which are clumps of cells that can turn into cancer over time.<br><br>Risk factors for developing colon cancer include an older age, African-American ethnicity, a personal history of colorectal polyps or cancer, inflammation of the bowel, a family history of colon cancer, low fiber and high fat diet, a sedentary lifestyle, obesity, smoking, alcohol, type 2 diabetes, and certain genetic syndromes.<br><br>Common symptoms can include changes in bowel habits (diarrhea, constipation, or narrowing of the stool) lasting for more than a few days, a feeling of needing to have a bowel movement, rectal bleeding, blood in the stool,	Colon cancer is a type of sickness that starts in the colon. The colon is the longest part of the large intestine, which is a part of your digestive system ‚Äî the system that processes the food and drink you take in, getting all the nutrients your body needs from them, and then gets rid of the so-called waste.<br><br>Think of your body like a city and colon cancer like a bad road that's not safe to drive on. Just like a city works to fix a bad road so cars can drive, doctors have ways to help fix the problem when someone has colon cancer.<br><br>This sickness happens when some cells in the colon start growing the wrong way. Instead of growing and splitting in an orderly manner, they grow and split faster than they should, causing problems. It's like if you had a plant that grew way too big and started taking over your garden.<br><br>Early on, colon cancer doesn't cause symptoms, but later on, it

© 2024 Musheyev D et al. *JAMA Network Open.*

Input	Free non-prompted Chat GPT	Free prompted ChatGPT	Paid non-prompted Chat GPT	Paid prompted ChatGPT
	Screening for colon cancer is recommended for individuals aged 50 to 75, or earlier for those with higher risk factors. Common screening methods include colonoscopy, where a flexible tube is inserted into the rectum to view the entire colon, and stool-based tests that check for blood or DNA related to colon cancer.<br><br>Treatment for colon cancer depends on the stage of the disease and may involve surgery, chemotherapy, radiation therapy, or a combination of these treatments. Earlier detection of colon cancer through screening can increase the chances of successful treatment and improve survival rates.	To find out if someone has colon cancer, a doctor will do some tests. One test is called a colonoscopy, where a small camera is used to look inside the colon to check for any abnormalities. If cancer is found, the doctor will discuss treatment options, such as surgery to remove the cancer or chemotherapy to help get rid of the cancer cells.<br><br>Remember, it's always important to be aware of your body and to tell an adult if you have any concerns. If detected early, colon cancer can be treatable, and there are ways to help prevent it too.	persistent abdominal discomfort, a feeling that your bowel doesn't empty completely, weakness or fatigue, and unexplained weight loss.<br><br>Diagnosis typically involves a colonoscopy, where a long flexible tube is inserted into the rectum and allows for visual inspection of the colon. A tissue sample or biopsy may be taken during a colonoscopy. If cancer is detected, doctors usually order further tests (like CT, MRI, or PET scans) to determine the extent or stage of the cancer.<br><br>Treatment options depend on the stage of the cancer and can include surgery, radiation therapy, chemotherapy, targeted therapies, or immunotherapy. Colon cancer is better prevented than treated; hence regular screening is important to identify early stages or precancerous polyps. Changes in lifestyle and diet also help in prevention.	may cause things like tummy pain, blood in the poop, or weight loss without trying. That's why doctors often recommend colon cancer screening - a kind of health check-up - after a certain age. It's a way to spot and fix that bad road before it causes too many problems.<br><br>Always remember, if you have any questions or feel something isn‚Äôt right, don't hesitate to tell your parents or an adult you trust. They can help you find more answers or get any help you need.

© 2024 Musheyev D et al. *JAMA Network Open.*

eTable 3. Interpretation of Flesch-Kincaid Reading Grade Levels and Formula for Reading Ease

Reading Ease	Reading grade level	Verbal description	Percent of people likely to understand
0-30	college	Very difficult	4.5
30-50	13-16	Difficult	33
50-60	10-12	Fairly Difficult	54
60-70	8-9	Standard	83
70-80	7	Fairly Easy	88
80-90	6	Easy	91
90-100	5	Very Easy	93

Reading Ease is derived from the formula:

Reading Ease = 206.835 - (0.846) * (number of syllables per 100 words) - (1.015) * (average sentence length)

1. P. Ley & T. Florio (1996) The use of readability formulas in health care, Psychology, Health & Medicine, 1:1, 7-28, DOI: 10.1080/13548509608400003

© 2024 Musheyev D et al. *JAMA Network Open.*
