# Supplementary material for: Readability and Information Quality in Cancer Information From a Free vs Paid Chatbot
Source: JAMA Netw Open. 2024 Jul 26;7(7):e2422275. doi: 10.1001/jamanetworkopen.2024.22275 (PMC11282443; doi:10.1001/jamanetworkopen.2024.22275)
Supplement: Supplement 2. — Data Sharing Statement [file jamanetwopen-e2422275-s002.pdf]

Data Sharing Statement

Musheyev. Readability and Information Quality in Cancer Information from Free vs Paid ChatGPT. *JAMA Netw Open*. Published July 24, 2024.
doi:10.1001/jamanetworkopen.2024.22275

Data
Data available: Yes
Data types: Data (not involving human participants)
How to access data: Please email abdo.kabarriti@downstate.edu if you would like access to the data for research purposes.
When available: With publication

Supporting Documents
Document types: None

Additional Information
Who can access the data: Researchers whose proposed use of the data has been approved.
Types of analyses: For research purposes.
Mechanisms of data availability: with the investigator's support.
